# An Elusive Prize: Transcutaneous Near InfraRed Spectroscopy (NIRS) Monitoring of the Liver

**DOI:** 10.3389/fped.2020.563483

**Published:** 2020-11-19

**Authors:** Peter W. Guyon, Tara Karamlou, Kanishka Ratnayaka, Howaida G. El-Said, John W. Moore, Rohit P. Rao

**Affiliations:** ^1^Division of Pediatric Cardiology, University of California San Diego School of Medicine, Rady Children's Hospital, San Diego, CA, United States; ^2^Division of Pediatric Cardiothoracic Surgery, Cleveland Clinic Children's and the Heart Vascular and Thoracic Institute, Cleveland, OH, United States

**Keywords:** congenital heart disease, non-invasive monitoring, regional oximetry, cardiac output (CO), near-infra read spectroscopy, shock

## Abstract

**Introduction:** We postulate a relationship between a transcutaneous hepatic NIRS measurement and a directly obtained hepatic vein saturation. If true, hepatic NIRS monitoring (in conjunction with the current dual-site cerebral-renal NIRS paradigm) might increase the sensitivity for detecting shock since regional oxygen delivery changes in the splanchnic circulation before the kidney or brain. We explored a reliable technique for hepatic NIRS monitoring as a prelude to rigorously testing this hypothesis. This proof-of-concept study aimed to validate hepatic NIRS monitoring by comparing hepatic NIRS measurements to direct hepatic vein samples obtained during cardiac catheterization.

**Method:** IRB-approved prospective pilot study of hepatic NIRS monitoring involving 10 patients without liver disease who were already undergoing elective cardiac catheterization. We placed a NIRS monitor on the skin overlying liver during catheterization. Direct measurement of hepatic vein oxygen saturation during the case compared with simultaneous hepatic NIRS measurement.

**Results:** There was no correlation between the Hepatic NIRS values and the directly measured hepatic vein saturation (*R* = −0.035; *P* = 0.9238). However, the Hepatic NIRS values correlated with the cardiac output (*R* = 0.808; *P* = 0.0047), the systolic arterial blood pressure (*R* = 0.739; *P* = 0.0146), and the diastolic arterial blood pressure (*R* = 0.7548; *P* = 0.0116).

**Conclusions:** Using the technique described, hepatic NIRS does not correlate well with the hepatic vein saturation. Further optimization of the technique might provide a better measurement. Hepatic NIRS does correlate with cardiac output and thus may still provide a valuable additional piece of hemodynamic information when combined with other non-invasive monitoring.

## Introduction

Patients admitted to the pediatric cardiovascular intensive care unit (CVICU) are at high risk of developing shock, a leading cause of cardiac arrest in children. Early recognition of shock is associated with expedited treatment and improved outcomes while delayed recognition leads to end organ dysfunction, including profound neurologic injury, and mortality (1, 2). Current CVICU hemodynamic monitoring relies on a combination of invasive and non-invasive methods to detect shock. Non-invasive methods are favored in children due to technical problems and size restraints (3). There is an unmet need for superior non-invasive hemodynamic monitoring systems.

Transcutaneous Near Infra-Red Spectroscopy (NIRS) technology allows for non-invasive, real-time monitoring of regional tissue oxygenation saturation (rS0_2_). This technology has demonstrated utility in pediatric critical care, especially in the perioperative period, and many centers in the United States consider dual-site organ centric NIRS monitoring (brain and kidney) an important component of Intensive Care Unit (ICU) care (4). Dual-site NIRS monitoring can be particularly helpful during shock, when regional blood flow redistributes to maximize oxygen delivery to the brain and heart (5), because changes in local cardiac output to organs will likely result in changes in their measured tissue oxygenation. Nevertheless, dual-site NIRS monitoring provides an incomplete picture, since decreases to gut flow precede significant decreases to kidney or brain flow (5).

The addition of NIRS monitoring to the hepatic vascular bed would theoretically help capture important changes in oxygen distribution to the splanchnic circulation during the time leading up to and including clinical shock. (In the acute setting, sudden changes in regional tissue oxygen saturation are more likely due to increased oxygen extraction from the tissues owing to decreased regional cardiac output, as opposed to a change in the intrinsic metabolism of the specific organ). In that way, changes in the rS0_2_ value might signal changes in blood flow to that organ. Thus, the addition of hepatic NIRS monitoring to the existing two-site paradigm could theoretically reveal invaluable new insights into the regional oxygenation and redistribution of blood flow in low cardiac output states, facilitating earlier goal directed interventions and improved patient outcomes.

Before any rigorous test of the above hypothesis can be undertaken, a reliable technique for hepatic NIRS monitoring must be established. NIRS generally offers a heavily venous-weighted proxy for the oxygenation of the tissue under the probe, so it could be expected to correlate with hepatic vein saturation (although this might not necessarily be the case, given the unique circulation of the liver, as we discuss below.) Previous attempts to measure hepatic rSO_2_ by NIRS have provided mixed results, potentially due to multiple factors, including outdated NIRS technology and excessive surface artifact (6–12). The following prospective pilot study tested a novel technique for non-invasive transcutaneous NIRS monitoring of regional hepatic oxygenation by comparing NIRS measurements to a simultaneous hepatic vein saturation obtained during a cardiac catheterization.

## Method

### Patient Selection, Population

**Figure 1:**
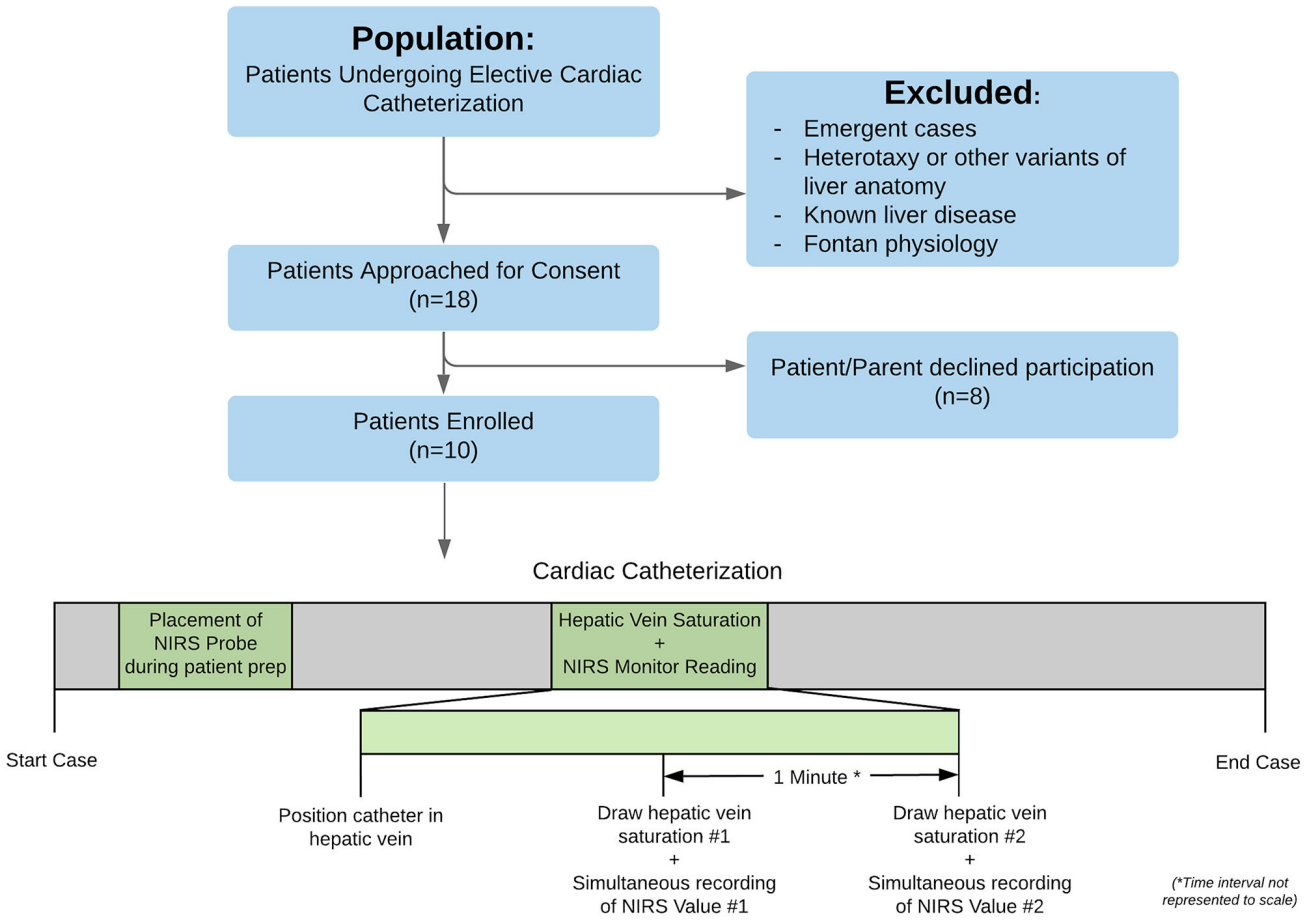
Workflow chart of the project, demonstrating screening, consent, and enrollment. Once enrolled, patients underwent their cardiac catheterization as normal, with the additional research protocol steps highlighted in green: (1) placement of the NIRS probe during patient preparation and (2) positioning of a catheter in the hepatic vein (confirmed by fluoroscopy) with subsequent drawing of two small blood samples spaced 1 min apart.

An outline of the overall study design, along with a timeline, is shown in Figure 1. Patients already scheduled for cardiac catheterization by the interventional cardiology team were screened for eligibility. Patients undergoing emergent catheterizations, patients with heterotaxy or other known anatomical variants of liver anatomy, or patients with known liver disease were excluded. “Known liver disease” was defined as any one of the following: (a) evidence of hepatic fibrosis on biopsy; (b) elevated transaminase level on labs obtained within the previous 3 months; (c) chronic vascular congestion of the liver as evidenced by enlarged liver by palpation/percussion on physical exam within the previous 6 months; (d) other primary liver disease documented in medical record. Patients with Fontan physiology were also excluded as they were presumed to have some underlying liver disease. Patients not excluded after review were approached for enrollment in the study and offered informed consent in compliance with the principles and practices stipulated by the Institutional Review Board. All ten patients enrolled in the pilot study completed the study protocol.

### Description of Technique

**Figure 2:**
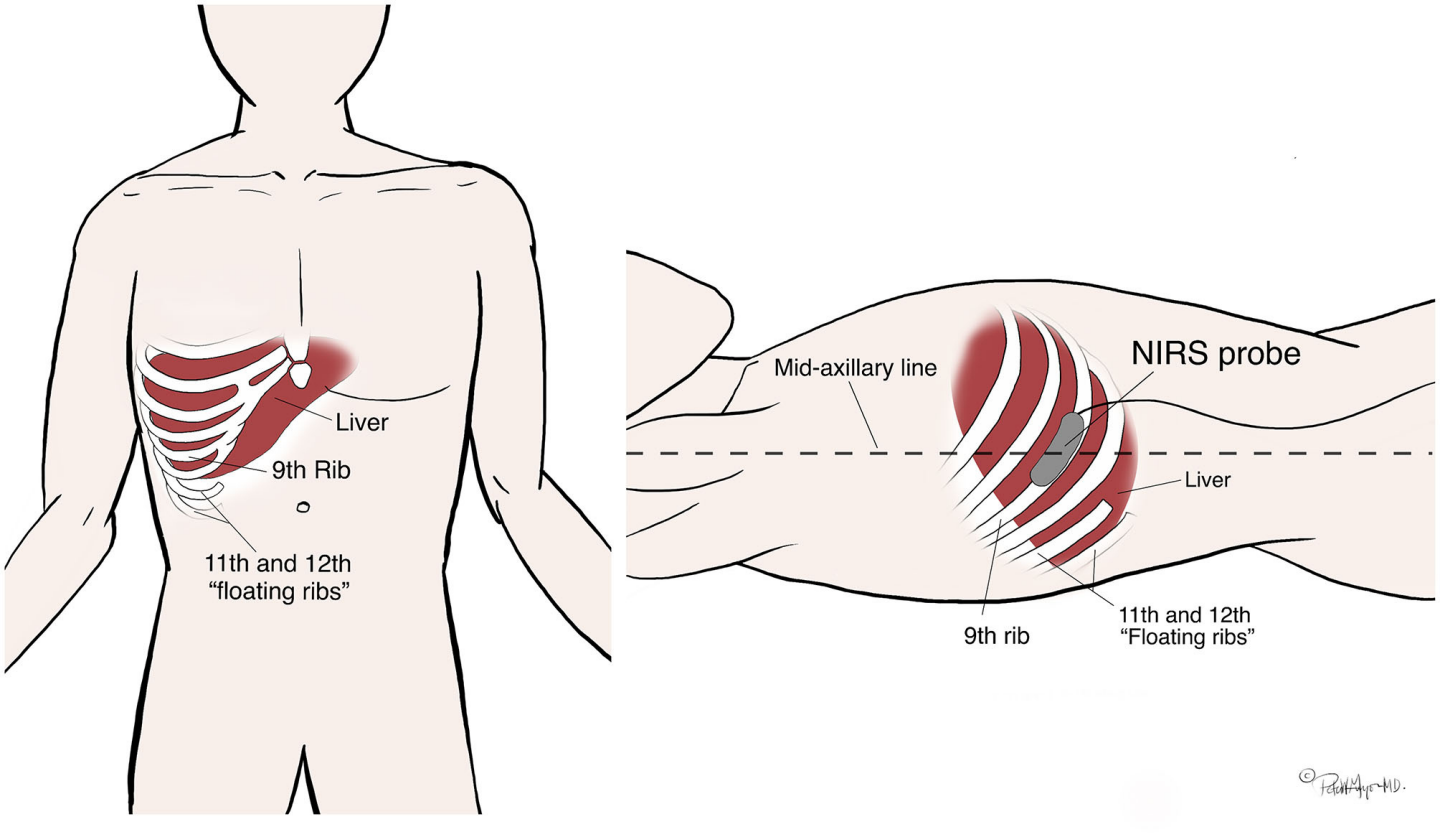
Reproducible technique for NIRS probe placement. The probe location was identified by first palpating the inferior right costal margin and following laterally around to near the mid-axillary line (the tenth rib); then feeling for one rib superior (the ninth rib.) The NIRS probe was then placed at the mid-axillary line over the ninth rib, running parallel to the path of the rib along the cranial aspect to avoid the neurovascular bundle which runs under the caudal aspect.

All typical institutional cardiac catheterization practice was followed including general endotracheal anesthesia. During patient preparation, a study team member placed one Cerebral/Somatic Oximetry Sensor (INVOS 5100C; Somanetics Corporation, Troy, MI) on the patient's skin overlying the superior angle of the ninth right rib in the midaxillary line. The sensor has one light emitting diode and two detectors (730 nm and 810 nm wavelengths) with inter-optode distances of 3 cm and 4 cm, providing an optical field with a depth of 2 cm beneath the skin. The probe was placed running parallel atop the rib (theoretically allowing a “window” through the less-vascularized bone and into the underlying liver tissue) (See Figure 2). Then per normal cardiac catheterization procedure the interventional cardiologist established vascular access. During the right heart catheterization, a catheter was positioned in the hepatic vein, (confirmed by fluoroscopy and/or a small hand injection angiogram), and two blood samples of 0.1–0.2 cc volume were drawn, separated by at least 1 min, and no more than 2 min. When each blood sample was obtained, a study team member simultaneously documented the NIRS monitor (rS0_2_-H) measurement, the invasive arterial blood pressure, the heart rate, and the arterial saturation by pulse oximetry. The blood samples were immediately processed by a portable oximeter in the catheterization lab by the trained staff, and the hepatic venous saturations (SvH0_2_) were recorded. Additional recorded procedural variables included: height, weight, body surface area (BSA), cardiac output (Fick method or thermodilution method), cardiac index, Qp:Qs, invasive oximetry saturations (central venous, pulmonary artery, systemic.) The patient's primary diagnosis as well as past medical and surgical history were also recorded.

### Data Analysis

Ten patients were enrolled. The specific aim of the study was to validate a technique for transcutaneous hepatic NIRS monitoring as a surrogate for hepatic vein saturation. We compared simultaneous hepatic NIRS values (rS0_2_-H) with hepatic vein blood saturation values (SvH0_2_) obtained during the catheterization. rS0_2_-H was defined as the average of the two hepatic NIRS measurements and SvH0_2_ was defined as the average of the two invasive hepatic vein saturation measurements. Scatterplots were created among all numeric variables to explore relationships between factors. Simple correlational analysis and descriptive statistics appropriate for the normality and type of data were utilized.

## Results

A summary of basic patient demographic data as well as correlation analysis is provided in Table 1. The median patient age was 6.4 years (range 1.9–18.9 years) and the median weight was 28.0 kg (range 10.4–100.7 kg). The underlying diagnoses were variable, and in general, reflected the most prevalent populations undergoing elective cardiac catheterization at our center. These included atrial septal defect (*N* = 2), orthotopic heart transplant (*N* = 4), patent ductus arteriosus (*N* = 2), double outlet right ventricle (*N* = 1), and one patient with pulmonary stenosis and partial anomalous pulmonary venous return.

**Table 1:** Demographic and clinical factors tested for correlation with hepatic NIRS.

	**(*N*)**	**Mean**	**Standard deviation**	**Median**	**IQR**	**Pearson correlation coefficients** **Prob >|r| under H0: Rho = 0**
Hepatic vein saturation (%)	10	60	14.5	66	47.3–70.6	−0.035
						0.924
Weight (kg)	10	36	29.7	28.0	12.9–48.4	0.622
						0.055
Height (cm)	10	123.5	40	120.5	87–157	0.772
						0.009
Body surface area (m^2^)	10	1.08	0.61	0.96	0.56–1.46	0.705
						0.023
Age at catheterization (years)	10	8.2	7.0	6.5	2.1–14.5	0.632 0.050
Central venous pressure (mmHg)	10	5.9	1.5	6.0	5.3–7.0	0.175 0.629
Cardiac index (L/min/m^2^)	10	3.8	1.28	4.1	2.8–4.7	−0.353
						0.317
Cardiac output (L/min)	10	3.5	1.36	3.3	2.4–4.1	0.808
						0.005
Mixed venous saturation (%)	9	75	5.6	77	75–78	0.115
						0.768
Arterial blood saturation (%)	8	96	3.7	98	95–98.3	0.016
						0.971
Systolic pressure (mmHg)	10	77	10.8	76	75–78	0.739
						0.015
Diastolic pressure (mmHg)	10	43	7.6	40	38–47	0.755 0.012
Hemoglobin (mg/dL)	10	14.5	5.9	12.6	12.2–13.6	−0.040
						0.912
Heart rate (beats per minute)	10	89	19.1	95	83–99	−0.534
						0.112

**Figure 3:**
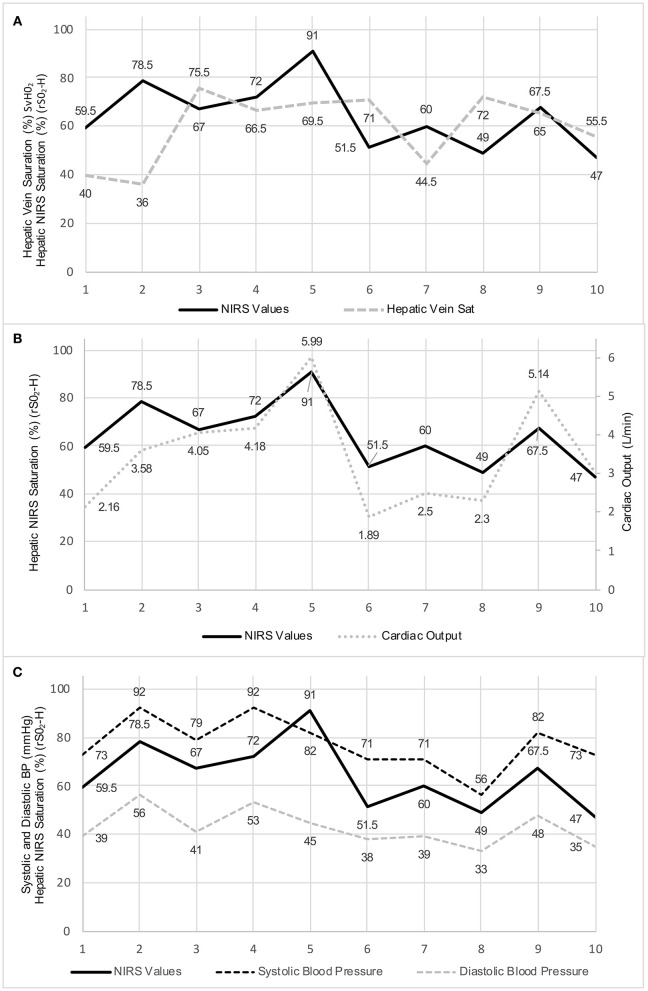
**(A)** Graphical representation of the hepatic NIRS measurements and the hepatic vein saturation values for the ten study patients. **(B)** Graphical representation of the hepatic NIRS measurements and the cardiac output values for the ten study patients. **(C)** Graphical representation of the hepatic NIRS measurements and the systolic and diastolic blood pressure values for the ten study patients.

Correlations between hepatic NIRS value and the various factors are also shown in Table 1. There was no correlation between the Hepatic NIRS values and the directly measured hepatic vein saturation (*R* = −0.035; *P* = 0.9238). There was also no correlation between the Hepatic NIRS values and the central or mixed venous saturation (it should be noted in most cases, this was an SVC and not an IVC saturation value) (*R* = 0.1105; *P* = 0.7682). However, the Hepatic NIRS value was strongly correlated with the cardiac output (*R* = 0.808; *P* = 0.0047), the systolic arterial blood pressure (*R* = 0.739; *P* = 0.0146), and the diastolic arterial blood pressure (*R* = 0.7548; *P* = 0.0116), indicating that it may reflect systemic perfusion over a discrete range of blood pressure measurements (see Figures 3A–C for a graphical representation of these relationships.) Interestingly, Hepatic NIRS was also related to the height (*R* = 0.772; *P* = 0.009), body surface area (*R* = 0.705; *P* = 0.023), and was borderline statistically significant for relationship to age at intervention (*R* = 0.632; *P* = 0.050).

## Discussion

We found that using a technique placing the NIRS probe directly over and running parallel to the ninth right rib at the mid-axillary line, there is no correlation between the reported rS0_2_-H value and the directly measured SvH0_2_. We also found no correlation between the reported rS0_2_-H value and the central or mixed venous saturation. These findings are important since they demonstrate that there might be significant difficulty in obtaining a reliable non-invasive measure of the regional oxygen saturation of the liver. At the same time, we found unexpectedly that the Hepatic NIRS value was strongly correlated with the cardiac output and systemic blood pressure, indicating that while the measurement may not accurately reflect the saturation of the hepatic vein, it may still contain important hemodynamic information. While we admit the small numbers and design of this pilot study do not allow for a full exploration of these results, in the remaining discussion we offer theoretical reasons for these findings, which can serve as a springboard toward future studies.

### Negative Primary Endpoint: No Correlation With Hepatic Vein Saturation and NIRS Measurement

The concept of multisite NIRS monitoring to characterize changes in integrative circulatory physiology has been previously described and has been extensively evaluated in the cerebral (13–18), splanchnic (8, 19), and quasi- global circulations (9, 20, 21). Therefore, it is reasonable to accept that the theory and technology underlying NIRS monitoring is sound and unlikely the reason for our inability to correlate our NIRS measurement with the hepatic vein oxygen saturation. With this in mind, the most vexing question is: what was the NIRS monitor actually measuring?

NIRS technology uses a modified Beer- Lambert principle to measure the relative concentrations of oxy and deoxy-hemoglobin species, yielding an assessment of tissue oxygenation by measuring the color of oxygen in the blood circulating through the optical field beneath the probe. Unlike pulse oximetry, which evaluates the pulsatile arterial flow, NIRS devices evaluate the entirety of the optical field, including the non-pulsatile optical component. In most organs, the resulting measurement is venous-weighted and predictable, since the pulsatile component is artery/arteriole and the non-pulsatile component is venule/vein. However, the liver is unique in its microcirculatory anatomy and physiology.

The hepatic afferent vascular supply is dual, split between the splanchnic supply from the portal vein and the systemic arterial supply from the hepatic artery (~70:30 vein:artery under physiologic conditions) (22). Hepatic arterioles and portal venules converge and communicate just prior to emptying into the sinusoid, the functional capillary of the liver where oxygen and other nutrients are exchanged with hepatocytes and toxins are removed (22). As opposed to a gradual narrowing from arteriole to capillary, there is a more sudden transition from arteriole to sinusoid thought to be maintained by sphincter-like specialized structures (22). This anatomy matters because arterial blood changes suddenly from a pulsatile to a non-pulsatile pattern before delivering oxygen to the tissues. Theoretically, a higher cardiac output and/or systemic blood pressure driving more arterial blood through the sphincter-like structures would lead to a higher saturation in the non-pulsatile sinusoids, resulting in a higher NIRS value, and one that would not necessarily correlate with the true hepatic vein saturation. This construct would agree with our findings. It should be noted that currently it is unclear exactly how these sphincter cells perform to auto-regulate arterial flow at the microcirculatory level.

Another explanation for the lack of correlation between our NIRS measurements and hepatic vein saturations is due to heterogeneity of the venous and arterial supply to different portions of the liver. Using contrast agents injected into the portal venous system, Timm and Vollmar found some areas of liver which seemed to have almost no portal venous input (23), which confirmed earlier reports of heterogeneity of portal venous blood supply (24–27). For this reason, it is possible that any given area directly under the NIRS probe might be an area which over-or-under-represents the venous component of the liver supply and thus will not give an accurate reading of the true global hepatic vein saturation. Since it is not possible to know which segment might have lower or higher portal venous contribution, it might never be possible to measure a reliable surrogate of portal vein saturation using surface NIRS.

In contrast to the theoretical explanations offered above, a simpler explanation to our negative primary endpoint might be a product of our specific technique for NIRS probe placement. Our technique was based around the idea that placement of the NIRS probe overlying the rib would be optimal because the bone would provide a “window” of relatively under-vascularized tissue, allowing the NIRS monitor to gather a pure signal from the underlying hepatic tissue. However, flat bones including ribs, are active sites of hematopoiesis. It is possible that the signal from various erythrocyte precursor cells, etc., which are the byproduct of bone marrow activity, (even if the volume of such tissue is relatively small) could have an out-sized effect on the reported NIRS signal. Thus, it might be helpful in future studies to position the probe so as to minimize the amount of rib in line with the NIRS signal, placing the probe perpendicular to the rib rather than parallel. Additionally, although we used physical exam findings to place our NIRS probe (as outlined above), we did not use ultrasound or other imaging to identify the exact location of the liver or its depth beneath our probe. We posit that for most non-obese pediatric patients, a depth of 2–3 cm below the skin should provide a window to the liver tissue, but these findings need further study to be confirmed.

### Valuable Information

Although our study was negative for our primary outcome, the correlation with cardiac output (as well as blood pressure) we report suggests possible utility for hepatic NIRS in concert with other oximetry data for non-invasive cardiac output monitoring. Tibby et al. have shown that using only clinical variables (before widespread use of NIRS) including heart rate, blood pressure, central venous pressure, urine output, capillary refill, and serum lactate, even experienced ICU providers could not reliably estimate cardiac output in children (28). Direct invasive measurement of cardiac output relies on dilution techniques (dye dilution, thermodilution, lithium dilution) or direct Fick measurements (utilizing O2 and C02 spirometry, mass spectrometry), each with their own set of limitations and complications (29). Central venous catheter use, which is necessary in the more common thermodilution methods, is associated with both mechanical complications such as vessel perforation and thrombosis as well as infectious complications (30–32), the rates of which are likely higher in pediatric patients (33).

Given the risks and complications associated with invasive monitoring, less invasive methods of estimating cardiac output (34) are theoretically superior, especially in pediatric patients. NIRS has the advantage of being completely non-invasive, generally easy to use, and well-studied. Currently, an arterial-venous oxygen difference (AV02 difference) compiled from pulse oximetry (proxy for arterial saturation) and somatic NIRS (proxy for mixed venous saturation) can be used as a general gauge of cardiac output in an intensive care unit. Even if a hepatic NIRS measurement cannot be relied upon as a surrogate for a hepatic vein saturation (and hopefully with further refinements to technique it can), it might still be valuable as an additional real-time trend for the cardiac output in concert with cerebral and somatic NIRS monitoring, offering an additional piece of the hemodynamic puzzle.

### Limitations

The most obvious limitation of our study was the small number of patients enrolled that limits the confidence is any statistical estimates. Potential inaccuracies inherent to liver physiology and due to our placement of the NIRS monitor have been discussed above and will need to be accounted for in future studies with more patients. Additionally, we did not use ultrasound to measure the depth of liver tissue in relation to our pobe. In future studies, we would consider adding this step to our methods to ensure that the liver tissue is being interrogated by the NIRS monitor. We would also consider using a NIRS instrument with adjustable interoptode distances, which might provide more depth of tissue penetration and a better signal. An additional factor is the heterogeneity of our study population in terms of age, size, and underlying congenital heart disease. We attempted to keep the exclusion criteria limited in the hopes that this technique would be widely applicable. However, in future studies we may attempt to limit our population in order to protect against confounding which is inherent in a heterogeneous study population. Finally, multiple NIRS and hepatic vein saturation measurements throughout the catheterization, as well as cerebral and renal NIRS values for the different time points, would allow for a more robust analysis and the exploration of longitudinal trajectories and trends. These data would help establish a more concrete connection between cardiac output and hepatic NIRS, but go beyond the objectives of this limited pilot study, and acquisition of multiple blood samples in pediatric patients often pose regulatory hurdles for study completion.

### Conclusions

In a pilot study of a novel technique for transcutaneous NIRS monitoring of the hepatic vascular bed, no correlation was found between the NIRS measurements and a direct sample of hepatic venous blood during a cardiac catheterization. Interestingly, the hepatic NIRS measurement was strongly correlated to some clinical factors, including cardiac output (although there was no correlation with cardiac index), and systemic blood pressure. Further study is needed to better understand the correlation with these hemodynamic parameters and whether or not NIRS monitoring of the liver can provide relevant data in a clinical setting. Finally, it is possible that iterative refinement to the technique based on our preliminary findings might still produce an accurate measurement of the hepatic vein saturation.

## Data Availability Statement

The original contributions presented in the study are included in the article/supplementary materials, further inquiries can be directed to the corresponding author/s.

## Ethics Statement

The studies involving human participants were reviewed and approved by Institutional Review Board The University of California, San Diego. Written informed consent to participate in this study was provided by the participants' legal guardian/next of kin.

## Author Contributions

RR conceived the project idea and edited the manuscript. PG designed the project workflow, obtained permission from the IRB, directed patient consent, directed data collection, and was primary author of the manuscript. TK directed the statistical analysis and edited the manuscript. KR, HE-S, and JM aided data collection, guided project design, and edited the manuscript. All authors contributed to the article and approved the submitted version.

## Conflict of Interest

The authors declare that the research was conducted in the absence of any commercial or financial relationships that could be construed as a potential conflict of interest.
